# p53 activation vs. stabilization: an acetylation tale from the C-terminal tail

**DOI:** 10.18632/oncoscience.534

**Published:** 2021-05-07

**Authors:** Ning Kon, Wei Gu

**Affiliations:** ^1^ Institute for Cancer Genetics, Vagelos College of Physicians and Surgeons, Columbia University, New York, NY 10032, USA; ^2^ Department of Pathology and Cell Biology, Vagelos College of Physicians & Surgeons, Columbia University Irving Medical Center, New York, NY 10032, USA; ^3^ Herbert Irving Comprehensive Cancer Center, Vagelos College of Physicians & Surgeons, Columbia University Irving Medical Center, New York, NY 10032, USA

**Keywords:** p53, acetylation, activation, tumor suppression

**Figure 1:**
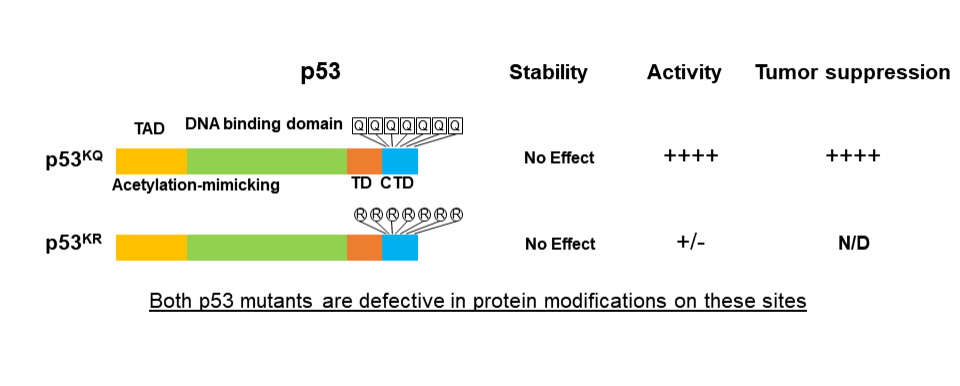
**Figure 1: A model for p53 acetylation in regulating its transactivation, stability and tumor suppression.** The effects of acetylation mimicking, lysine to glutamine (K→Q) or lysine to arginine (K→R, for simply blocking protein modification) mutations at the C-terminus on the protein stability, transactivation, and tumor suppressor function of p53 are summarized. TAD: transactivation domain, yellow; TD: tetramerization domain, red; CTD: C-terminal domain, blue. DNA binding domain is in green.

Tumor suppressor p53 is regarded as the guardian of genome because of its critical role in DNA damage responses [1]. One of the prominent features of the p53-mediated DNA damage response is the rapid accumulation of p53 protein partially due to the dissociation of p53 from Mdm2 and the increased p53 stability, followed by the transcription of p53 target genes to promote cellular functions to maintain genome integrity. In addition, p53 transcriptional activities can also be enhanced through posttranslational modifications, such as acetylation and phosphorylation. Numerous studies indicate that upregulation of p53 transcriptional activities through acetylation/deacetylation represents a dynamic process of p53 regulation under normal physiological settings such as development, aging related conditions, and tumor suppression [2]. Notably, the C-terminus of p53 harboring a lysine-rich basic domain is thought to be the major inhibitory domain because of varies different types of protein modification, interacting with repressors as well as other properties such as non-specific DNA binding. Acetylation of the lysine residues in the C-terminal domain has been shown to counteract other types of protein modification such as ubiquitination, to repel inhibitors such as SET [2], and to augment p53 transcriptional activities by enhancing sequence-specific DNA binding and recruiting transcriptional activators [3]. Indeed, p53 acetylation at different sites apparently accommodates the need of promote-specific and diverse activation of p53 functions, compared to the transient and fast response required to combat the DNA damage. To understand the precise role of p53 C-terminal acetylation *in vivo*, we generated the acetylation-mimicking *p53^KQ^* mice and studied the phenotypes associated with the mutant p53 in the absence of stress. More importantly, the tumor suppressor function of the mutant p53 was examined in a pancreatic ductal adenocarcinoma (PDAC) mouse model. Consistent to the previous studies, mimicking acetylation of the C-terminus of p53 enhanced its activities as a transcription factor, indicated by the increased expression of p53 target genes in the mutant *p53* mice. Specifically, a number of key target genes were activated in *p53^KQ/KQ^* embryos during embryonic development, leading to deficient brain development and perinatal lethality [2]. Some of the targets were induced even in *p53^KQ/-^* mice, leading to hematopoiesis failure and premature death in *p53* mutant mice [4]. Notably, although the amount of p53 mutant protein dictated when and in which tissue the p53 target genes were induced, there was no robust p53 stabilization in affected tissues, suggesting p53 activation and p53 stabilization can be regulated independently. Interestingly, previous studies showed that in contrast to the acetylation mimicking mutant, simply blocking other types of protein modifications by lysine to arginine substitutions at these same sites had very mild effects on p53 activity *in vivo* [5, 6] (Figure 1). Taken together, these findings indicate that acetylation but not simply blocking other types of modification such as ubiquitination, at the C-terminus, is critical for activating p53-mediated tumor suppression in vivo, particularly when p53 stabilization is not operative.

Importantly, expression of p53 KQ mutant in the PDAC mouse model (*arffl/fl,k-ras,pdx1-cre*) resulted in delayed tumor progression, consistent with the tumor suppressor function of p53 upon its activation [4]. In addition, we also deleted *sirt1* in the same PDAC mouse model, because Sirt1 has been shown to deacetylate p53 to regulate the acetylation levels of endogenous p53. The results indicated that although sirt1 deletion in the PDAC mouse model (*sirt1fl/fl,arffl/fl,k-ras,pdx1-cre*) did not allow the mice to survive as long as the p53^KQ^–expressing PDAC mice (*p53KQneo/KQneo,arffl/fl,k-ras,pdx1-cre*), it did delayed the tumor progression significantly (data not shown). These results are understandable because the extent of acetylation levels in the absence of SirT1 at the endogenous p53 C-terminus are likely less than 100% due to the presence of other deacetylases. Nevertheless, these results underscore the potential use of Sirt1 inhibitors or other types of HDAC inhibitors in suppression of tumor progression in the tumors retaining the wild type p53.

Recently, the C-terminal mutations of p53 were reported in human patients, which result in loss of all acetylation sites [7]. Remarkably, the two patients displayed similar phenotypes observed in *p53^KQ^* mice, such as microcephaly and brain malfunctions, hematopoiesis failure at early age. Despite of these abnormalities, the patients are not susceptible to cancer. The study went on to show that the human p53 C-terminal truncation mutant also had augmented transcriptional activities, similar to the mouse p53 C-terminal truncation mutant [8, 9]. Even though the mouse p53 C-terminal truncation mutant is almost identical to the human p53 mutant, the phenotypes in mouse p53 C-terminal truncation homozygous mutant are much weaker than the phenotypes in human patients, in whom the mutations are presented as heterozygote (the mutations are *de novo*) [8, 9]. These findings suggest that the C-terminus of p53 may have greater effects in human than in mouse, further validating the importance of acetylation in p53 regulation in human cancers. Thus, modulation of p53 acetylation levels represent a new aspect for the p53-based therapy in human cancers.
